# A Systematic Review of Barriers to Breast Cancer Care in Developing Countries Resulting in Delayed Patient Presentation

**DOI:** 10.1155/2012/121873

**Published:** 2012-08-22

**Authors:** Ketan Sharma, Ainhoa Costas, Lawrence N. Shulman, John G. Meara

**Affiliations:** ^1^Program in Global Surgery and Social Change, Harvard Medical School, 25 Shattuck Street, Boston, MA 02115, USA; ^2^Department of Plastic and Oral Surgery, Children's Hospital Boston, 300 Longwood Avenue, Enders 1, Boston, MA 02115, USA; ^3^Duke University School of Medicine, 201 Trent Drive, Durham, NC 27715, USA; ^4^Dana Farber Cancer Institute, Brigham and Women's Hospital, 450 Brookline Avenue, Boston, MA 02115, USA

## Abstract

*Background*. Within the developing world, many personal, sociocultural, and economic factors cause delayed patient presentation, a prolonged interval from initial symptom discovery to provider presentation. Understanding these barriers to care is crucial to optimizing interventions that pre-empt patient delay. *Methods*. A systematic review was conducted querying: PubMed, Embase, Web of Science, CINAHL, Cochrane Library, J East, CAB, African Index Medicus, and LiLACS. Of 763 unique abstracts, 122 were extracted for full review and 13 included in final analysis. *Results*. Studies posed variable risks of bias and produced mixed results. There is strong evidence that lower education level and lesser income status contribute to patient delay. There is weaker and, sometimes, contradictory evidence that other factors may also contribute. *Discussion*. Poverty emerges as the underlying common denominator preventing earlier presentation in these settings. The evidence for sociocultural variables is less strong, but may reflect current paucity of high-quality research. Conflicting results may be due to heterogeneity of the developing world itself. *Conclusion*. Future research is required that includes patients with and without delay, utilizes a validated questionnaire, and controls for potential confounders. Current evidence suggests that interventions should primarily increase proximal and affordable healthcare access and secondarily enhance breast cancer awareness, to productively reduce patient delay.

## 1. Introduction

 Breast cancer remains the most common cancer and most common cause of cancer-related mortality among women worldwide [1]. While incidence rates have historically been higher in the developed world, there has been a recent sharp increase in incidence and mortality in the developing world [2]. Furthermore, the case-fatality rate (or relative survival, approximated as the compliment of the mortality to incidence ratio [3]) within these nations tends to be lower, largely due to patients presenting at more advanced stages [2]. 

Delayed patient presentation refers to a prolonged interval between discovery of initial symptom to presentation to a provider and is typically defined as greater than 12 weeks, as periods longer than this have been associated with poorer survival [4]. In contrast, provider delay refers to an extended interval from initial patient presentation to effective oncological treatment. Nonetheless, providers and health systems may serve as primary sources of health-related education and affect patient delay as well. Patient delay has been associated with increased tumor size, more advanced stage at presentation, and poorer long-term survival [5] and is a significant concern in developing countries. For example, one study in sub-Saharan Africa found that 90% of breast cancer patients presented with stage III or IV disease, exhibited a median primary tumor size of 10 cm, and displayed clinically palpable nodal disease [6], a pattern of disease so advanced that even optimal Western therapy would offer minimal survival value. As a result, future interventions aimed at controlling the increase in global breast cancer burden should address delayed patient presentation in addition to extending access to advanced care.

 Numerous factors may result in delayed patient presentation and can be conceptually organized into three potentially overlapping categories: personal, sociocultural, and economic (Table 1); an understanding of these factors is crucial to optimizing future interventions that preempt patient delay. Existing research on this topic has mostly focused on the developed world: one systematic review of 19 such studies concluded there was strong evidence for older age and moderate evidence for not attributing initial symptom to breast cancer, not disclosing breast symptoms to another, discovering an initial symptom other than a lump, nonwhite ethnicity, and fewer years of education [4]. 

 Unfortunately, similar research focusing on developing countries is more limited. As there may exist substantial geographical variation in barriers to care due to differing social, cultural, and economic contexts—especially between richer developed countries and poorer developing ones—findings from developed countries cannot be generalized to developing ones. To our knowledge, a systematic review of barriers to care resulting in patient delay of breast cancer within developing nations has not been performed. Therefore, the purpose of this study is to (1) systematically review the current available research examining personal, sociocultural, and economic variables that may result in delayed patient presentation within developing countries and (2) highlight areas of deficiency requiring further research such that subsequent interventions may be streamlined and personalized according to the developing country's context of need.

## 2. Materials and Methods

 This systematic review was modeled on the PRISMA guidelines [7]. Database inquiry was initiated into PubMed using the Mesh headings “Patient Acceptance of Health Care”, “Delayed Diagnosis”, and “Breast Neoplasms” with a text search of “delay” or “late” in titles and abstracts. This search was expanded and modified into the following additional databases: Embase, Web of Science, CINAHL, Cochrane Library, J East, CAB, African Index Medicus, and LiLACS. Additional relevant studies were identified by manually examining bibliographies of included articles and querying content experts.

 All studies required approval by Institutional Review Boards (IRBs). Cross-sectional studies, case series, and case-control analyses were acceptable for inclusion (Table 2).

Studies had to include a definition of delayed patient presentation or report the interval between initial symptom and presentation, define potential barriers to care, and statistically associate how putative variables correlate to delay or that interval. The primary outcome of interest was delayed patient presentation; for studies without their own definition, delay was defined as symptom to presentation interval >12 weeks. Furthermore, studies had to be conducted in developing countries, defined as a low- or middle-, income economy by the World Bank based on per capita GNI [8]. To maintain contemporary relevance, only papers published after 1990 were included. 

 Exclusion criteria were as follows: studies that did not occur within developing countries, occurred before 1990, did not explicitly state what barriers to care were inquired about in their questionnaire/survey, did not consistently apply the same questionnaire/survey to every patient, did not include a specific definition of delayed patient presentation and/or did not report symptom-presentation interval, investigated sources of provider delay only, consisted of cohort studies, randomized-controlled trials (RCTs), expert consensus/opinion, or other reviews, had a sample size of *n* < 10, or included only males or patients with benign breast pathology.

Authors Sharma and Costas independently reviewed abstracts and (if necessary) full articles to determine inclusion eligibility, with disagreements resolved by discussion. Sharma abstracted study information into tables, with both checking information for accuracy. Sharma and Costas independently assessed study quality and then compared results, with disagreements resolved by discussion.

 Quality was graded using criteria adapted from the United States Preventive Services Task Force (USPSTF). For each study, Sharma and Costas graded selection bias, measurement bias, and confounding potential as good (G), fair (F), or poor (P). Ratings were converted into numerical values as follows: G = 2, F = 1, and P = 0. Then, a composite score evaluating each study's overall risk of bias (internal validity) was averaged from these components, with 1.5 or higher considered good (G), 1.0 to 1.49 considered fair (F), and less than 1.0 considered poor (P). Finally, each study's external validity was evaluated.

 After data abstraction and quality assessment, this study's authors met for discussion about results and continued iterative review until reaching consensus about key message and conclusions. No meta-analyses could be performed due to heterogeneity of study populations, differences in inquiry regarding specific barriers to care, and differences in definition of delayed presentation.

## 3. Results

### 3.1. Search Results

**Figure 1:**
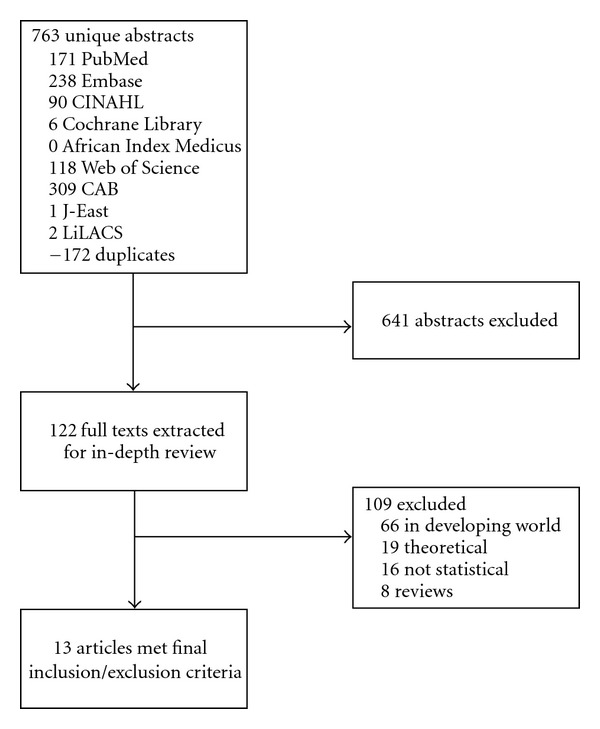
Study selection flowchart.

Overall, 13 articles were included in final analysis, all identified via primary literature search (Figure 1).

Included studies occurred in the following developing countries: Aziz et al. [9] and Malik et al. [10] in Pakistan, Ezeome [11] and Ukwenya et al. [12] in Nigeria, Harirchi et al. [13] and Montazeri et al. [14] in Iran, Abdel-Fattah et al. [15] in Egypt, Ali et al. [16] in India, Clegg-Lamptey et al. [17] in Ghana, Landolsi et al. [18] in Tunisia, Norsa'adah et al. [19] in Malaysia, Piñeros et al. [20] in Colombia, and Thongsuksai et al. [21] in Thailand. 11 of the 13 were cross-sectional analyses, whereas 2 were case series examining patients with delay only (Table 3).

### 3.2. Selection Bias

 Eight studies were rated “Good” or posed low potential for selection bias: Abdel-Fattah et al. [15], Ali et al. [16], Ezeome [11], Harirchi et al. [13], Landolsi et al. [18], Montazeri et al. [14], Norsa'adah et al. [19], and Piñeros et al. [20]. These were relatively high-powered, recruited from one or more high-volume centers, and included a wide array of patients with varying demographics. 

 Four studies were rated “Fair” or posed moderate potential for selection bias. Aziz et al. [9] was relatively high-powered (*n* = 286) but did not report pertinent demographic data. Malik et al. [10] (*n* = 103), Thongsuksai et al. [21] (*n* = 94), and Ukwenya et al. [12] (*n* = 99) reported demographic data but had medium-sized samples. The remaining study, Clegg-Lamptey et al. [17], was rated “Poor” for selection bias potential as it was relatively low-powered (*n* = 66).

### 3.3. Measurement Bias

 All 13 studies utilized standardized questionnaires applied consistently to each patient. In some cases, these questionnaires were validated by independent experts and tested in focus groups. These procedures reduce the potential for measurement bias. However, all studies assessed delay retrospectively, as patients were asked to remember the time interval from first symptom to presentation, introducing potential recall bias. In fact, there is evidence that patients may underreport duration of delay [22]. But this bias is acceptable and unavoidable, as delay must be assessed retrospectively for ethical reasons. Thus, twelve of the studies were evaluated as “Fair” or introducing moderate potential for measurement bias.

The remaining study, Norsa'adah et al. [19], was evaluated as “Poor” or introducing high potential for measurement bias, as they reported an outcome of “diagnosis delay” that combined patient and provider delay, defined as greater than 6 months from initial symptom to pathological diagnosis. Thus, potential barriers to care may be affecting provider delay as well, with independent effect on patient delay alone being less observable.

### 3.4. Confounding Potential

 An independent estimate of how a potential barrier-to-care may delay patient presentation requires controlling for confounding, including other potential barriers to care and patient demographic data. Since it is unethical to randomize patients to potential barriers to care and then prospectively assess their delay, statistically rigorous multivariate regression is required for effective covariate control.

 Four of the studies were evaluated as “Good” as they included numerous covariates into such a regression model: Ali et al. [16], Montazeri et al. [14], Norsa'adah et al. [19], and Thongsuksai et al. [21]. Another study was evaluated as “Fair” as it introduced moderate potential for confounding: Abdel-Fattah et al. [15] utilized multivariate regression but controlled for only age and education status, two of many potential confounders. The remaining eight studies were all evaluated as “Poor” as they employed univariate analyses only. 

### 3.5. Overall Risk of Bias/Internal Validity

 Ultimately, two studies were classified as “Good” for posing low overall risk of bias: Ali et al. [16] in India and Montazeri et al. [14] in Iran. Both employed large sample sizes, included a variety of patients with different demographics, utilized standardized questionnaires, and controlled for many potential confounders via multivariate regression. 

 Another seven studies were classified as “Fair” or posed moderate overall risk of bias: Abdel-Fattah et al. [15], Ezeome [11], Harirchi et al. [13], Landolsi et al. [18], Norsa'adah et al. [19], Piñeros et al. [20], and Thongsuksai et al. [21]. These studies contribute meaningful information by highlighting certain barriers to care as potentially causing patient delay. Nonetheless, their observed results should be interpreted with some caution.

 The remaining four studies posed high overall risks of bias and were classified as “Poor”: Aziz et al. [9], Clegg-Lamptey et al. [17], Malik et al. [10], and Ukwenya et al. [12]. These studies involved low or medium sample sizes and did not attempt to control for confounders. Thus, any barriers to care found to be significant via these studies must be interpreted with caution, especially if they did not exhibit significance via the “Good” or “Fair” studies.

### 3.6. Qualitative Synthesis

 A qualitative synthesis utilizing only the “Good” and “Fair” studies is provided in Table 4. Results from “Good” studies are listed as exhibiting good or strong evidence and “Fair” studies as exhibiting far or moderate evidence. Furthermore, barriers to care are classified into a personal, sociocultural, or economic schematic.

 Overall, studies rated as “Good” showed evidence that positive family history of breast cancer, lower education level, lower income status, and being unmarried/divorced/widowed contributes to delayed patient presentation.

Studies rated as “Fair” showed evidence for a number of barriers to care. In terms of personal characteristics, older age, marriage, negative family history of breast cancer, and clinical presentation specifics (namely, presence of breast ulcer, lack of pain, and presence of palpable axillary lymph nodes) are moderately linked to patient delay. In terms of sociocultural context, alternative treatment use (including use of other practitioners and use of prayer), breast cancer awareness (including failure to practice breast self-exam and ignorance of initial symptoms as relating to cancer), and fear of treatment are moderately linked to patient delay. In terms of economic variables, rural residency and health systems context (especially lack of affiliation with a health system and lower access to physicians) are moderately linked to patient delay.

Notably, different studies yielded different and, sometimes, contradictory results. For example, one study found an association between being married and patient delay, while another observed an association between not being married and patient delay. In addition, one study found a positive correlation between family history and patient delay, while another observed a negative correlation instead. 

## 4. Discussion 

Delay is a major contributor to advanced-stage presentation of breast cancer [22], the predominant cause of poorer survival within the developing world [23]. Delayed presentation can be divided into two sequential components: patient delay, a prolonged interval from discovery of initial symptom to presentation to a qualified medical provider, and provider delay, a prolonged interval from patient presentation to first oncologic treatment. This systematic review evaluated the current research investigating potential personal, sociocultural, and economic barriers to care that may cause patient delay within the developing world (Table 1).

The predominant theme emerging from the results of this review emphasizes how poverty constitutes the underlying common denominator and most important barrier contributing to delayed patient presentation in these settings. These conditions of poverty are supported by evidence from both “Good” and “Fair” studies and chiefly manifested economically via lower income status, lesser education level, rural residency, and lack of access to healthcare systems (Table 4). This may reflect how these low-income developing countries “share an economic status” where “the infrastructure and human resources for cancer prevention or control are nonexistent or very limited in quantity, quality, and accessibility [24].” 

The association between the personal characteristics of marital status and family history with patient delay showed contradictory results from different studies. As noted, two studies associated being widowed or divorced with patient delay [14, 16], whereas another reported increased delay amongst married women [13]. In addition, one study observed a positive correlation between family history of breast cancer and patient delay [16], while another reported a negative correlation [13]. These conflicting results may be secondary to the varying risks of bias within the studies (Table 2). However, they may also reflect geographic variation in barriers to care due to the multidimensional heterogeneity of the developing world, where societies, cultures, and governments differ tremendously. As it remains important not to generalize barriers to care from the developed world over to the developing world, it remains similarly prudent not to extrapolate from one part of the developing world to another.

Importantly, all of the studies discovering a significant association between a sociocultural barrier-to-care and patient delay were classified as either “Poor” or “Fair.” This may imply that such variables are less influential, more variable from site to site, and/or harder to elucidate on investigation. However, neither of the “Good” studies inquired about specific sociocultural variables pertaining to breast cancer, such as stigma of disease, breast self-exam use, and fear of treatment. Therefore, while the current evidence for such sociocultural factors remains moderate, this may reflect the current paucity of overall “Good” studies, and future research is required to ascertain the true relationship between sociocultural variables and patient delay in the developing world. 

This review's findings are somewhat different than a prior review focusing on patients in the developed world [4]. Here, Ramirez et al. concluded there was strong evidence that patient delay was associated with older age and unrelated to patient marital status, and there was moderate evidence that patient delay was associated with fewer years of education, nonwhite ethnicity, discovery of initial symptom other than a breast lump, not disclosing breast symptoms to another, and not attributing breast symptoms to cancer [4]. 

As stated, this review observed variable risks of bias amongst included studies. Unfortunately, most studies were of poor to fair internal validity, such that results must be concluded with caution. The “Good” studies [14, 16] followed several strategies which should serve as guidelines for future investigations in this arena. First, selection bias should be minimized by using either a high-powered cross-sectional examination or a matched case-control analysis, as a comparison between patients with and without delay reveals causative factors that may result in delay. Second, measurement bias in retrospective studies should be minimized by utilizing a independentlyvalidated and standardized questionnaire or survey, and by explicitly defining patient delay as separate from provider delay. Third, confounding should be addressed via statistically rigorous multivariate regression that controls for patient demographics and other barriers to care.

This review does suffer from certain limitations. First, while the search was thorough, involving 760+ initial abstracts from 9 English and foreign databases, there is a chance that other articles published in foreign languages and/or foreign journals that are less accessible through large databases were missed. Second, the qualitative synthesis incorporated only barriers to care that exhibited statistical significance in “Good” or “Fair” studies. This strategy importantly accounts for study quality, but also prioritizes statistical significance in a “Fair” study over nonsignificance in a “Good” study, largely due to the high burden to prove statistical significance. Furthermore, this strategy neglects results from the “Poor” studies if they are not also corroborated by “Fair” or “Good” studies. While the “Poor” studies certainly contribute valuable information in their respective countries, they ultimately posed too high a risk of bias for their results to be considered independent and reliable.

The results from this review should guide future interventions that target patient delay within the developing world. Specifically, since poverty appears to be the most common and strongest barrier-to-care, future interventions should primarily attempt to enhance access to proximate and affordable healthcare. Traveling away from home, family and work responsibilities, and high cost of diagnosis and treatment constitute particularly burdensome problems facing the poor. In addition, while the evidence for sociocultural barriers may be less strong as of now, future interventions should also attempt to raise breast cancer awareness and reduce stigma of disease, as secondary objectives. Notably, causes of delay may also be hierarchical; for instance, if poverty is successfully addressed, other factors may play a more significant role. This review suggests that these tactics may productively pre-empt patient delay and begin to control the burden of breast cancer within the developing world, as long as interventions are specificallytailored to the country of need.

## 5. Conclusion

 Understanding the array of personal, sociocultural, and economic barriers to care that contribute to delayed patient presentation of breast cancer within developing countries is crucial to controlling the disease globally. This systematic review found strong evidence for personal and economic factors relating to patient delay and moderate evidence for sociocultural ones. Future research is required specifically to developing countries and/or regions that is high-powered, utilizes a standardized questionnaire, and controls for potential confounders. Nonetheless, existing evidence suggests that interventions targeting these barriers to care should primarily extend access to affordable and proximate healthcare, and secondarily increase breast cancer awareness, to productively reduce patient delay.

## Figures and Tables

**Table 1:** Potential barriers-to-care causing delayed patient presentation.

Personal	Sociocultural	Economic
Age Ethnicity Marriage status Clinical presentation Personal history Family history	Breast cancer awareness: knowledge of symptoms, breast self-exam (BSE) use Alternative treatment use: other practitioners, prayer, herbal remedy Fear of examination, treatment Stigma of disease Denial/anxiety Overall level of social support	High cost of medical care High cost of travel to clinic Obligations at home/work Access to health systems Location of origin Education level Income status

**Table 2:** PICOTTS eligibility criteria for studies included in review.

	Inclusion	Exclusion
Population	Female patients with breast cancer living within developing countries.	Males, benign breast disease, living in the developed world.
Intervention	A questionnaire inquiring about personal, sociocultural, and economic variables that may have contributed to delayed patient presentation.	Any study that did not explicitly define potential variables contributing to patient delay, or did not apply questionnaire consistently to every patient.
Control	Patients who presented without delay, but not required.	None.
Outcome	A definition of patient delayed presentation or an interval >12 weeks between discovery of first symptom to presentation to a provider.	Any study that did not include its own definition of patient delay, did not report a symptom-presentation interval, or examined provider delay only.
Time (intervention)	Any study conducted after 1990.	Any study conducted before 1990.
Time (follow-up)	No follow-up period required	None.
Study design	Case series, cross-sectional, or case-control, with a sample size of at least *n* = 10.	Single case report, cohort study, randomized-controlled trial, expert consensus and/or other review, any sample size of *n* < 10.

**Table 3:** Critical appraisal of included studies.

Author	Country (*n*)	Design	Factors relating to patient delay		Selection bias	Measurement bias	Confounding potential	Internal validity	External validity
Abdel-Fattah et al. [15]	Egypt *n* = 565	Cross-sectional	Failure to practice BSE		Good	Fair	Fair	Fair	Fair
								
Ali et al. [16]	India *n* = 522	Cross-sectional	Lower education level Lower income status	Unmarried, widowed, or divorced	Good	Fair	Good	Good	Good
									
Aziz et al. [9]	Pakistan *n* = 286	Cross-sectional	Lower annual income		Fair	Fair	Poor	Poor	Poor
								
Clegg-Lamptey et al. [17]	Ghana *n* = 66	Case series	Financial incapability Fear of mastectomy Herbal treatment	Prayer Ignorance	Poor	Fair	Poor	Poor	Poor
									
Ezeome [11]	Nigeria *n* = 164	Cross-sectional	Alternative practitioner use		Good	Fair	Poor	Fair	Fair
			Prayer house use						
									
Harirchi et al. [13]	Iran *n* = 200	Cross-sectional	Lesser access to physicians Negative family history Ignorance of symptoms Lower level of education Rural residence	Older age Married Lower economic status Less importance attributed to BSE	Good	Fair	Poor	Fair	Fair
									
Landolsi et al. [18]	Tunisia *n* = 160	Cross-sectional	Nonattribution of symptoms to cancer	Lack of BSE use	Good	Fair	Poor	Fair	Fair
									
Malik et al. [10]	Pakistan *n* = 103	Cross-sectional	Rural residency Reluctance to see doctors	Poverty	Fair	Fair	Poor	Poor	Fair
									
Montazeri et al. [14]	Iran *n* = 190	Cross-sectional	Widowed/divorced Lower education level	Positive family history	Good	Fair	Good	Good	Fair
									
Norsa'adah et al. [19]	Malaysia *n* = 328	Cross-sectional	Use of alternative therapy Initial symptom: breast ulcer Ignorance of symptoms as breast cancer	Fear of treatment Presence of palpable axillary lymph nodes	Good	Poor	Good	Fair	Good
									
Piñeros et al. [20]	Colombia *n* = 903	Cross-sectional	Poorer housing conditions Lack of pain as symptom Ignorance of symptoms as breast cancer	Older age Lower education level Lack of affiliation with health system	Good	Fair	Poor	Fair	Good
									
Thongsuksai et al. [21]	Thailand *n* = 94	Cross-sectional	Unmarried		Fair	Fair	Good	Fair	Fair
								
Ukwenya et al. [12]	Nigeria *n* = 99	Case series	Alternative treatment use Ignorance of symptoms as breast cancer Fear of mastectomy	High cost of treatment Family refused hospital treatment	Fair	Fair	Poor	Poor	Fair

**Table 4:** Qualitative synthesis of barriers-to-care utilizing good and fair studies.

Evidence strength	Barriers-to-care		
	Personal	Sociocultural	Economic
Good (strong)	Unmarried, widowed, or divorced		Lower income status
	Positive family history		Lower education level

Fair (moderate)	Older age Married Negative family history Clinical presentation: ulcer, lack of pain, palpable axillary lymph nodes	Alternative treatment use: other practitioners, prayer Breast cancer awareness: failure to practice BSE, ignorance of symptoms as cancer Fear of treatment	Rural residency Health systems: lack of affiliation, lesser access to physicians
